# Dehydroepiandrosterone exerts antiglucocorticoid action on human preadipocyte proliferation, differentiation, and glucose uptake

**DOI:** 10.1152/ajpendo.00314.2012

**Published:** 2013-09-10

**Authors:** Joanne C. McNelis, Konstantinos N. Manolopoulos, Laura L. Gathercole, Iwona J. Bujalska, Paul M. Stewart, Jeremy W. Tomlinson, Wiebke Arlt

**Affiliations:** Centre for Endocrinology, Diabetes, and Metabolism, School of Clinical and Experimental Medicine, University of Birmingham, Birmingham, United Kingdom

**Keywords:** dehydroepiandrosterone, human adipogenesis, 11β-hydroxysteroid dehydrogenase type 1, insulin sensitivity

## Abstract

Glucocorticoids increase adipocyte proliferation and differentiation, a process underpinned by the local reactivation of inactive cortisone to active cortisol within adipocytes catalyzed by 11β-hydroxysteroid dehydrogenase type 1 (11β-HSD1). The adrenal sex steroid precursor dehydroepiandrosterone (DHEA) has been shown to inhibit 11β-HSD1 in murine adipocytes; however, rodent adrenals do not produce DHEA physiologically. Here, we aimed to determine the effects and underlying mechanisms of the potential antiglucocorticoid action of DHEA and its sulfate ester DHEAS in human preadipocytes. Utilizing a human subcutaneous preadipocyte cell line, Chub-S7, we examined the metabolism and effects of DHEA in human adipocytes, including adipocyte proliferation, differentiation, 11β-HSD1 expression, and activity and glucose uptake. DHEA, but not DHEAS, significantly inhibited preadipocyte proliferation via cell cycle arrest in the G1 phase independent of sex steroid and glucocorticoid receptor activation. 11β-HSD1 oxoreductase activity in differentiated adipocytes was inhibited by DHEA. DHEA coincubated with cortisone significantly inhibited preadipocyte differentiation, which was assessed by the expression of markers of early (*LPL*) and terminal (*G3PDH)* adipocyte differentiation. Coincubation with cortisol, negating the requirement for 11β-HSD1 oxoreductase activity, diminished the inhibitory effect of DHEA. Further consistent with glucocorticoid-opposing effects of DHEA, insulin-independent glucose uptake was significantly enhanced by DHEA treatment. DHEA increases basal glucose uptake and inhibits human preadipocyte proliferation and differentiation, thereby exerting an antiglucocorticoid action. DHEA inhibition of the amplification of glucocorticoid action mediated by 11β-HSD1 contributes to the inhibitory effect of DHEA on human preadipocyte differentiation.

glucocorticoid excess characteristically causes central obesity and insulin resistance. The local reactivation of glucocorticoids represents an important mechanism mediating glucocorticoid action. The enzyme 11β-hydroxysteroid dehydrogenase type 1 (11β-HSD1) converts inactive cortisone (E) to active cortisol (F) via its oxoreductase activity in a variety of tissues, importantly including liver and adipose tissue, which are major sites involved in the regulation of insulin sensitivity. The clinical significance of local glucocorticoid activation in the context of the metabolic syndrome has been demonstrated conclusively by a multitude of in vitro, in vivo, and clinical studies (12, 45). In addition, the selective inhibition of 11β-HSD1 activity is emerging as an exciting, novel therapeutic approach in type 2 diabetes and the metabolic syndrome (40, 42).

Several of the physiological effects of glucocorticoids oppose those of the adrenal steroid dehydroepiandrosterone (DHEA). DHEA and its sulfate ester DHEAS are the most abundant steroids in the human circulation and accumulate in adipose tissue at even greater concentrations (10–15 times circulating levels) (4, 17). DHEA is the principal sex steroid precursor in humans and can be converted directly to androgens, whereas its sulfate ester DHEAS first requires cleavage of the sulfate group by the enzyme steroid sulfatase (STS). In addition to its indirect role as a steroid precursor, DHEA can elicit direct effects in immune and vascular endothelial cells (35), although a specific receptor has as yet not been characterized. These studies have also highlighted distinct roles for DHEA and DHEAS (16, 38).

The metabolic effects of DHEA are complex, regulated by downstream metabolism of DHEA to sex steroids. Elevated circulating androgen levels are a cardinal feature of the common endocrinopathy polycystic ovary syndrome, which is associated with metabolic morbidities, including insulin resistance and abdominal obesity. Conversely, numerous murine-based studies have demonstrated that DHEA treatment has beneficial effects on whole body composition and several metabolic parameters, effects proposed to be attributable to its direct action on adipocyte biology, but also on other metabolic tissues such as muscle and liver. DHEA inhibits murine 3T3-L1 preadipocyte proliferation, differentiation, and ensuing lipid accumulation (20, 32, 34, 43), and consistently, murine in vivo studies have shown that DHEA retards fat accretion, reducing body fat percentage and mass, modulates circulating triglyceride levels, and attenuates hyperglycemia and/or hyperinsulinaemia (9–11, 18, 28). Rodent adrenals lack the ability to synthesize DHEA, and the validity of these findings to human physiology is questionable. Recently, it was shown that DHEA inhibits proliferation in PAZ6 preadipocytes, a cell line derived from human brown adipose tissue, and adipogenesis in omental but not subcutaneous preadipocytes (39), highlighting possible depot-specific effects of DHEA. However, human in vivo trials have proved less conclusive, and human in vitro studies are limited (44).

Interestingly, the expression and activity of 11β-HSD1 is inhibited by DHEA in murine adipocytes (2, 43) and rat liver (22), providing a potential mechanism by which the effects of DHEA oppose those of glucocorticoids. The aim of this study was twofold; first, provide a comprehensive analysis of DHEA metabolism in human adipocytes and the effects of DHEA and DHEAS on human adipogenesis, and second, examine the effect of DHEA on 11β-HSD1 in human adipocytes and resulting glucocorticoid reactivation based on our hypothesis that DHEA inhibits adipogenesis via 11β-HSD1.

## RESEARCH DESIGN AND METHODS

### Chub-S7 Cell Model Validation

The Chub-S7 cell line (Nestlé Research Centre, Lausanne, Switzerland) was derived from human subcutaneous adipose tissue by coexpression of human telomerase reverse transcriptase and papillomavirus E7 oncoprotein (HPV-E7) genes (14) and has previously been characterized in detail showing expression of typical white adipose tissue markers upon differentiation (6, 14, 19). Further characterization was undertaken to exclude a brown adipose tissue (BAT) phenotype as a result of rosiglitazone treatment during Chub-S7 differentiation (see below). We compared expression of the BAT marker uncoupling protein 1 (*UCP1*) (15) between Chub-S7 cells before and after differentiation, mature human subcutaneous adipocytes, and human primary-differentiated subcutaneous preadipocytes with or without the presence of DHEA and primary human hepatocytes as well as in primary mouse BAT, white adipose tissue, and hepatocytes. Subcutaneous human adipose tissue was obtained via the Human Biomaterials Resource Centre of the University of Birmingham from donors undergoing elective open abdominal surgery at the Queen Elizabeth Hospital Birmingham. All donors gave written informed consent as approved by the South Birmingham (UK) Research Ethics Committee. Tissue was immediately transferred from the operating theater to the laboratory in sterile containers, and mature adipocytes and preadipocytes were obtained after tissue digestion with collagenase (Sigma-Aldrich, St. Louis, MO) as described before (23). Primary mature adipocytes were left floating for 24 h in serum-free DMEM-F-12 before mRNA extraction. Primary preadipocytes were cultured in six-well plates in differentiation medium [containing DMEM-F-12, 10% fetal calf serum (FCS), 33 μM biotin, 17 μM pantothenic acid, 0.2 nM triiodothyronine (T_3_), 166 nM insulin, 45 mM methyl-3-isobutylxanthine, and 100 nM cortisol; all reagents were from Sigma] for 5 days and then in growth medium (DMEM-F-12, FCS, biotin, pantothenic acid, T_3_, insulin, and cortisol as before) until *day 14*, when mRNA was extracted. DHEA (100 nM or 10 μM) was added from *day 0* in treated cells. Primary human hepatocytes were obtained from Celsis (Brussels, Belgium), and mRNA was extracted after 24-h culture in proprietary medium (InVitroGRO; Celsis). Mouse tissues were obtained from BL6 wild-type mice kept in our laboratory, and mRNA was extracted from fresh tissues. All data are shown in mean arbitrary units (AU) ± SE.

### Cell Culture Conditions

Chub-S7 cells were maintained in DMEM-F-12 supplemented with 10% FCS. At 48 h postconfluence, differentiation was initiated by incubation with DMEM-F-12 supplemented with 33 μM biotin, 17 μM pantothenic acid, 0.2 nM T_3_, 167 nM insulin, 500 nM cortisol or cortisone, and 1 μM rosiglitazone for ≤21 days. On *day 7*, 14 and 21 photographs were taken to document cell morphology.

Human primary preadipocytes isolated from subcutaneous adipose tissue were obtained from Zen-Bio (Research Triangle Park, NC). Cells were maintained and differentiation induced according to the manufacturer's protocol. Briefly, preadipocytes were cultured to confluence in DMEM supplemented with 10% FCS and differentiation induced by incubation with proprietary differentiation medium (Zen-Bio) for 7 days. Cells were then cultured with proprietary adipose maintenance medium (Zen-Bio) for an additional 7 days.

### Proliferation Assays

#### Tritiated thymidine uptake assay.

Chub-S7 preadipocytes and human primary preadipocytes were seeded into a 24-well plate at densities 1 × 10^5^ and 2.5 × 10^5^ respectively. Following overnight culture, medium was supplemented with DHEA, androstenediol, or DHEAS (0–100 μM; Sigma-Aldrich). Following 24-, 48-, or 72 h incubation, cell proliferation was assessed by incubation with radiolabeled thymidine (0.2 μCi/well) for the final 6 h of culture. Proteins were precipitated with TCA, and cells were scraped in NaOH. The respective content of radiolabeled nuclear material in the resulting lysates was analyzed by scintillation counting. Data were expressed as percentage of control.

#### Colorimetric assay.

Chub-S7 preadipocytes were seeded into a 96-well plate (1 × 10^4^ cells/well). Following overnight culture, cells were pretreated for 2 h with the estrogen receptor antagonist faslodex, the androgen receptor antagonist flutamide (both 100 nM; Sigma-Aldrich), the glucocorticoid receptor RU-486 (5 μM; Sigma-Aldrich), cortisol, or cortisone (both 500 nM; Sigma-Aldrich) before treatment with DHEA (25 μM). At 24, 72, and 120 h, cell proliferation was assessed utilizing a nonradioactive cell proliferation assay (Promega, Madison, WI) according to the manufacturer's protocol. Luminescence was recorded at 490 nm utilizing a plate reader. Data were expressed as percentage of control.

#### Cell cycle analysis by flow cytometry.

Chub-S7 cells were seeded into a six-well plate (6 × 10^5^ cells/well). Following overnight culture, cells were incubated with ±25 μM DHEA. After 3 days, cells were incubated with propidium iodide (50 μg/ml) in phosphate citrate buffer containing 0.2 M Na_2_HPO_4_ and 0.1 M citric acid (24:1, pH 7.8, all Sigma-Aldrich) for 30 min. Samples were analyzed using a FACS IV flow cytometer at a wavelength of 488 nm. Approximately 10,000 cells per injection were analyzed.

### mRNA Expression Analysis

#### RNA extraction and RT.

RNA extraction was performed on Chub-S7 preadipocytes differentiated in the presence of DHEA (0–25 μM) for 7, 14, and 21 days as well as on the other tissues used in the Chub-S7 cell model validation experiment (see above). Total RNA was extracted using TRI-reagent (Ambion/Life Technologies, Grand Island, NY) according to the manufacturer's protocol.

#### Qualitative mRNA analysis.

Expression analysis of steroidogenic genes and steroid transporters was carried out using the gene-specific primers shown in Table 1. Amplifications were carried out at 95°C for 30 s, 60°C for 30 s, and 72°C for 30 s in a 20-μl final volume for 30 cycles.

**Table 1.** Qualitative mRNA analysis primers

Gene	Forward Primer	Reverse Primer
SULT2A1	CAGGAAGAACCATAGAGAAGATCTG	GTCTTACACAATGACCCCAGTC
STS	AGGACTTCCCACCGATGAGATTACCTTTG	AAAAGGGTCAGGATTAGGGCTGCTAGGAA
HSD11B1	ACCAGAGATGCTCCAAGGAA	ATGCTTCCATTGCTCTGCTT
OATP-A	CCACAAGATTTATATGTGGAAAATG	CATATATCCAGGTATGGCAGCC
OATP-B	CATGGGACCCAGGATAGGGCCA	GGCCTGGCCCCATCATGGTCACTG
OATP-C	GTTCAACCTGAATTGAAATCAC	GATGTGGAATTATATGTCCTACATGAC
OATP-D	GCTGAGAACGCAACCGTGGTTCC	GACTTGAGTTCAGGGCTGACTGTCC
OATP-E	GCCATGCCACTGCAGGGAAATG	TTCTGGTACACCAAGCAGGAGCCC
OATP-F	CAGAAAGACAATGATGTCC	CACATCTTTTAAATCCCCATTTGAGGC
OATP-8	GAATAAAACAGCAGAGTCAGCATC	GCAATATAGCTGAATGACAGG
OAT-4	CTCTGCGGTTTCCACAAACATGACC	CCACCATCAGTGTCAGTGAACTCAG
AKR1C3	ACTTCATGCCTGTATTGGGATTTG	CTGCCTGCGGTTGAAGTTTGATA
HSD17B4	AGTTCTCTCTCTTTCTTGTTGGCTCTGGA	GCGTCCTATTTCCTCAAATACAAAGGTACTCT
HSD3B1	GGAATCTGAAAAACGGCGGC	CTGAGATATAGTAGAACTGTCCTCGGATG
HSD3B2	GATCGTCCGCCTGTTGGTG	CTCTTCTTCGTGGCCGTTCTGGATGAT

SULT2A1, DHEA sulfotransferase; STS, steroid sulfatase; HSD11B1, 11β-hydroxysteroid dehydrogenase type 1; OATP, organic anion polypeptide; OAT-4, organic anion transporter 4; AKR1C3, 17β-hydroxysteroid dehydrogenase type 5; HSD17B4, 17β-hydroxysteroid dehydrogenase type 4; HSD3B, 3β-hydroxysteroid dehydrogenase.

#### Quantitative real-time mRNA analysis.

The mRNA expression of adipocyte differentiation markers [lipoprotein lipase (*LPL*), *G3PDH*, *FABP4*, and *UCP1*] and genes that regulate the local amplification of glucocorticoids (*HSD11B1*, *H6PDH*) was determined using an ABI 7500 sequence detection system (Applied Biosystems/Life Technologies). For the Chub-S7 model validation experiments, gene expression in Chub-S7 cells was analyzed on *days 0* (confluent, not differentiated) and *14* (differentiated). For all other experiments, gene expression was analyzed on *days 7*, *14*, and *21* in cells differentiated with cortisone and on *days 8* and *16* for cells differentiated with cortisol. Previous preliminary experiments confirmed that on *days 14* and *21* differentiated with cortisone and *days 8* and *16* differentiated with cortisol, cells had reached comparable differentiation states (assessed lipid accumulation and ΔC_T_ values of differentiation markers) due to the greater differentiation capacity of the active steroid.

Reactions were performed in 20-μl volumes on 96-well plates using 2× TaqMan Universal PCR Master Mix (Applied Biosystems/Life Technologies). Expression-specific probes and primers were supplied by Applied Biosystems as “assay on demand” reagents for the analysis of *LPL*, *G3PDH*, *SULT2A1*, *STS*, and *AKR1C3* and as proprietary primers for *FAB4* and *UCP1*. For analysis of *HSD11B1* and *H6PDH*, specific primers and probes were designed (Table 2). All reactions were normalized against the housekeeping gene 18S rRNA. Data were expressed as C_T_ values and used to determine ΔC_T_ values and fold changes using the following equation: fold increase = 2^−ΔΔC_T_^.

**Table 2.** Real-time mRNA analysis primers and probes

Gene	Forward Primer	Reverse Primer	Probe
HSD11B1	AGGAAAGCTCATGGGAGGACTAG	ATGGTGAATATCATCATGAAAAAGATTC	CATGCTCATTCTCAACCACATCACCAACA
H6PDH	CAGGTGTCCTAGTGCACATTGAC	GTAGCCCACTCTCTCGTCCAA	AAGGCACGCCCTCCCAGCG
GPD1 (G3PDH)	AGGGCCATCTGAAGGCAAACGCC	CCATCAGTTCATCGGCAAGAT	TCGTCTACCCCCTTAATAAGAGATATG

H6PDH, hexose-6-phosphate dehydrogenase; GPD1, glycerol-3-phosphate dehydrogenase.

### Enzyme Activity Assays

#### DHEA metabolism assay.

Chub-S7 cells were incubated in DMEM containing cold DHEA (20 nM) and tritiated DHEA (0.2 μCi/well) for 48 h. Following incubation, steroids were extracted using dichloromethane separated by thin-layer chromatography using n-hexane/1-hexanol (75:25; both Sigma-Aldrich) as the mobile phase system. Metabolites were identified by comigration with unlabeled reference steroids that were visualized by exposure to Lieberman-Burchard reagent (ethanol-acetic anhydride-sulfuric acid). Steroid conversion was quantified using a LabLogic AR-200 scanner (LabLogic, Sheffield, UK). Protein concentration was measured using a colorimetric 96-well plate assay (Bio-Rad, Hemel Hempstead, UK) and used to normalize conversion. Activity was expressed as percent conversion.

#### 11β-HSD1 activity assay.

Cells were cultured for 21 days in DMEM supplemented with DHEA. 11β-HSD1 oxoreductase and dehydrogenase activity were assessed as described previously (5). Briefly, cells were incubated with 100 nM cortisol or cortisone with appropriate tritiated tracer-[^3^H]cortisol (F) or [^3^H]cortisone (E) (0.02 μCi/reaction). Following incubation, steroids were extracted, separated, and quantified as above, utilizing chloroform and ethanol (92:8) as the mobile phase. Activity was expressed as cortisol or cortisone production in picomoles per milligram of protein per hour.

### Glucose Uptake Assay

Differentiated adipocytes were incubated with serum-free DMEM for 12 h prior to the addition of DHEA (final concentration 0–25 μM) for 2 h. Glucose uptake activity was assessed by measuring the uptake of 2-deoxy-d-[^3^H]glucose, as described previously (19). Analysis was performed in the presence or absence of 20 nM insulin. Data were expressed as percentage of control based on the measured disintegrations per minute.

### Statistical Analysis

Where data were normally distributed, the unpaired Student *t*-test was used to compare single treatments with control. If normality tests failed, then nonparametric tests were used. One-way ANOVA on ranks was used to compare multiple treatments, doses, or times (SigmaStat 3.1; Systat Software, Point Richmond, CA). Statistical analysis of real-time PCR data was performed on mean ΔC_T_ values and not transformed fold changes.

## RESULTS

### Chub-S7 Cell Model Validation

**Fig. 1.**
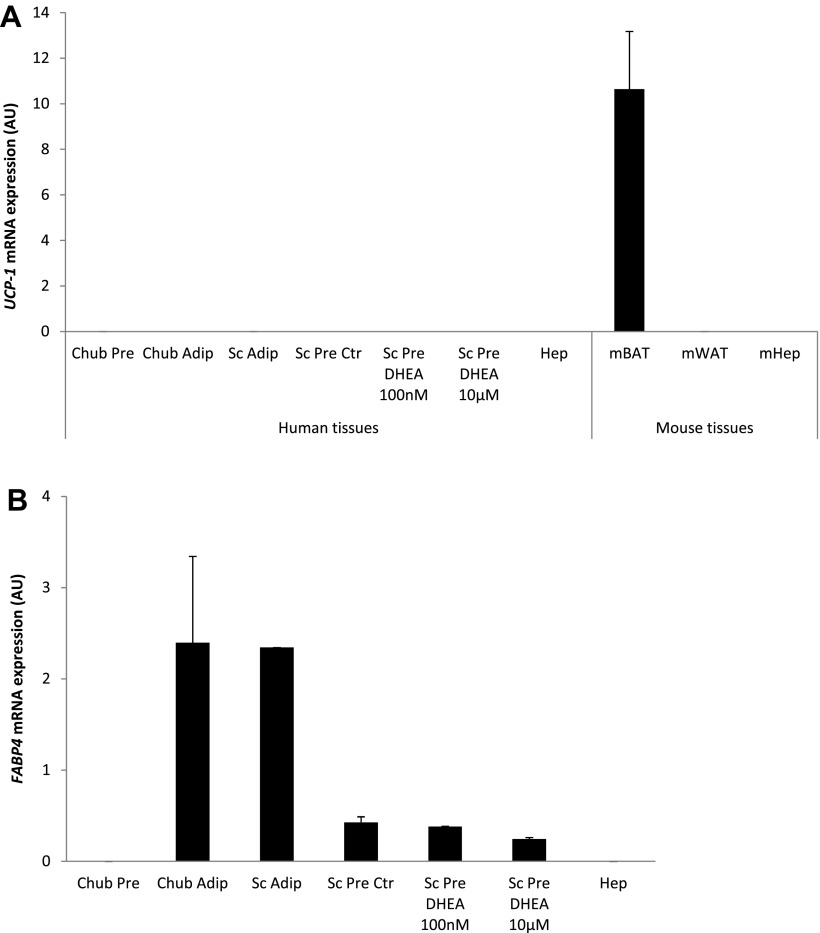
Chub-S7 cell line validation. *A*: quantitative real-time PCR mRNA expression of the brown adipose tissue (BAT) marker uncoupling protein 1 (*UCP1*) in Chub-S7 preadipocytes (Chub Pre; *n* = 3) and differentiated Chub-S7 adipocytes (Chub Adip; *n* = 3), mature human subcutaneous adipocytes (Sc Adip; *n* = 8), human primary differentiated preadipocytes without (Sc Pre Ctr; *n* = 3) or with dehydroepiandrosterone (DHEA) (Sc Pre DHEA; 100 nM and 10 μM, each *n* = 3), human primary hepatocytes (Hep; *n* = 4), mouse BAT (mBAT; *n* = 3), mouse white adipose tissue (mWAT; *n* = 4), and hepatocytes (mHep; *n* = 3). *B*: mRNA expression of the white adipose tissue marker fatty acid-binding protein 4 (*FABP4*) in Chub-S7 and human tissues as before. Chub-S7 cells, similarly to other non-BAT tissues, did not express any substantial amounts of *UCP1* but showed *FABP4* expression similar to white adipose tissue. Data expressed as mean arbitrary units (AU) ± SE from triplicate measurements. STS, steroid sulfatase.

Real-time mRNA expression of *UCP-1* in undifferentiated Chub-S7 cells was very low (0.00004 ± 0.00001 AU) and remained very low following differentiation (0.00002 ± 0.000004 AU) (Fig. 1*A*). In direct comparison, *UCP-1* expression in mouse BAT was 10-fold higher (10.6 ± 2.5 AU). Overall, *UCP-1* expression in Chub-S7 cells before and after differentiation was low comparared with the other non-BAT tissues examined, i.e., human mature subcutaneous adipocytes (0.00016 ± 0.00004 AU) and hepatocytes (0.00001 ± 0.00001 AU) (Fig. 1*A*). DHEA treatment of human primary preadipocytes did not have any effect on *UCP-1* expression (Fig. 1*A*).

The white adipose tissue marker *FABP4* was undetectable in undifferentiated Chub-S7 cells but was expressed at levels comparable with human mature adipocytes (2.4 ± 0.9 vs. 2.3 ± 0.0001 AU) following differentiation (Fig. 1*B*). Treatment of human primary preadipocytes with either 100 nM or 10 μM DHEA did not have any effect on *FABP4* expression (control: 0.43 ± 0.06 AU; 100 nM DHEA: 0.38 ± 0.003 AU; 10 μM DHEA: 0.24 ± 0.02 AU; Fig. 1*B*).

### DHEA Metabolism in Human Preadipocytes and Adipocytes

**Fig. 2.**
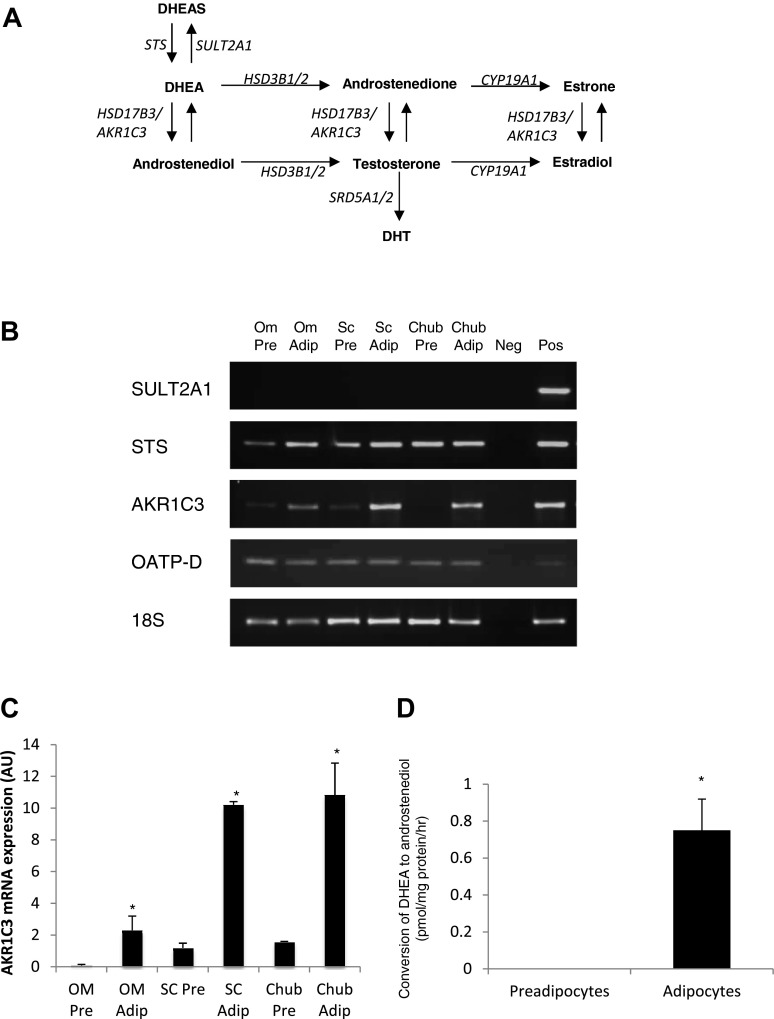
DHEA is metabolized to androstenediol in Chub-S7 adipocytes. *A*: schematic overview of key pathways of DHEA metabolism to DHEAS (sulfate ester of DHEA) and downstream steroids by steroidogenic enzymes. *B*: representative conventional PCR analysis of mRNA expression of steroidogenic genes in human omental preadipocytes (Om Pre) and adipocytes (Om Adip), Sc Pre and Sc Adip, and Chub Pre and Chub Adip. *C*: quantitative real-time PCR mRNA expression analysis of AKR1C3 in Om Pre and Om Adip, Sc Pre and Sc Adip, and Chub Pre and Chub Adip. Expression analysis confirmed the expression of AKR1C3 in Chub-S7 and human adipose tissue, which was increased in differentiated adipocytes relative to preadipocytes. Data are expressed as means ± SE of 3 independent triplicate experiments. *D*: Chub-S7 cells were incubated with [^3^H]DHEA for 24 h. Appreciable conversion of DHEA to androstenediol was observed in adipocytes but not proliferating preadipocytes (*P < 0.05 compared with preadipocytes).

Real-time mRNA expression analysis of key DHEA-metabolizing enzymes (Fig. 2*A*) in Chub-S7 cells and human subcutaneous primary adipocytes revealed no expression of *SULT2A1*, indicating that DHEA cannot be inactivated by sulfation to DHEAS within preadipocytes or adipocytes (Fig. 2*B*). In contrast, abundant expression of STS, which catalyzes the opposing reaction, was detected in all samples (Fig. 2*B*). We identified selective expression of *OATP-D*, an influx transporter of hydrophilic DHEAS (Fig. 2*B*), suggesting that DHEAS can actively enter the adipocyte.

Whereas mRNA expression of *HSD3B1* or *HSD3B2* was not detected, we observed expression of *AKR1C3*, encoding 17β-HSD type 5, in all samples (Fig. 2*B*). Quantitative mRNA expression analysis revealed that expression of *AKR1C3* was relatively low in preadipocytes but greatly increased following adipocyte differentiation (Fig. 2*C*).

Functional assays utilizing radiolabeled DHEA revealed that, consistent with the above finding, there is significant conversion of DHEA to androstenediol in fully differentiated adipocytes (Fig. 2*D*). In contrast, there was no appreciable conversion of DHEA in preadipocytes (Fig. 2*D*). Interestingly, despite the detection of mRNA transcripts encoding STS in both preadipocytes and mature adipocytes, we did not detect any conversion of DHEA and DHEAS, demonstrating a lack of functional activity of STS (data not shown).

### Effects of DHEA on Preadipocyte Proliferation

**Fig. 3.**
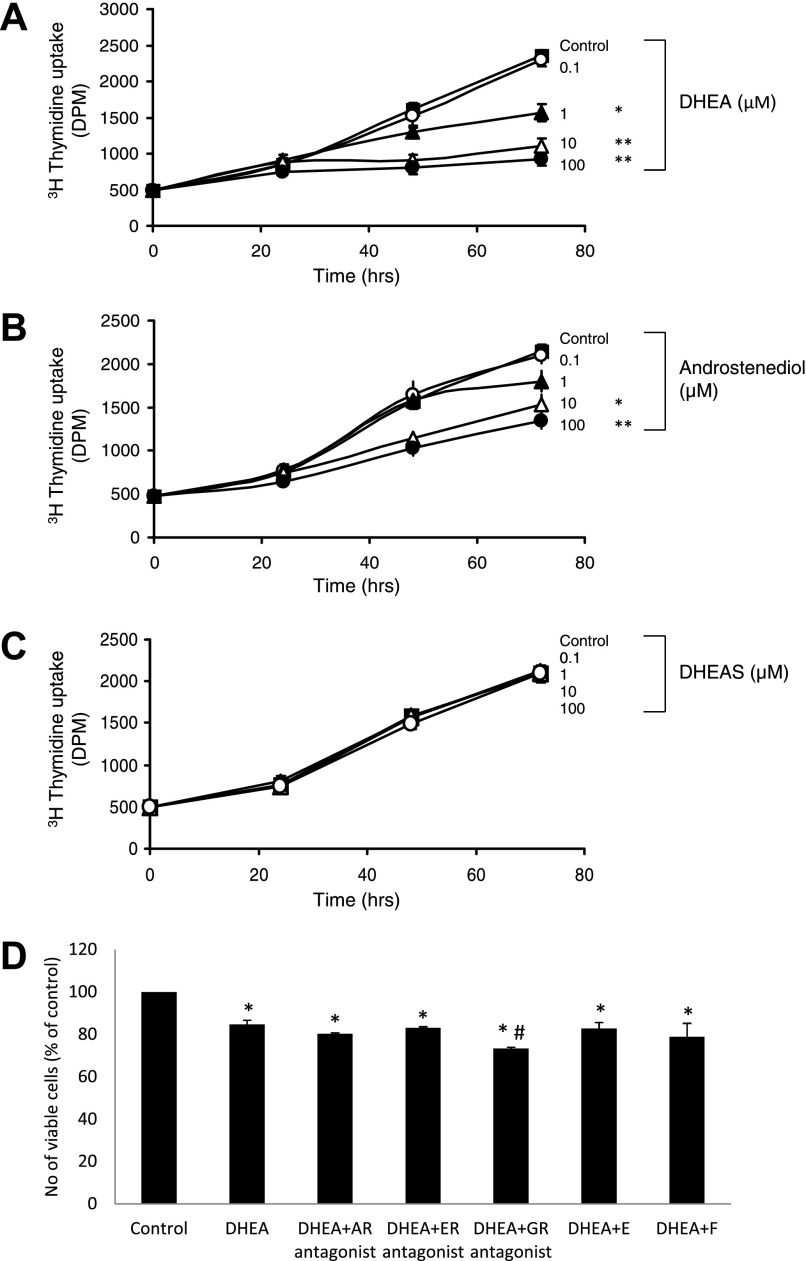
Dose-dependent inhibition of Chub-S7 preadipocyte proliferation by DHEA and androstenediol. Subconfluent Chub-S7 preadipocytes were incubated with DHEA, androstenediol, or DHEAS (0–100 μM) for 24, 48, or 72 h. *A* and *B*: proliferation was analyzed by incubation with 0.2 μCi of [^3^H]thymidine for the last 6 h of culture. *C*: DHEA (≥1 μM; *A*) and androstenediol (≥10 μM; *B*) significantly inhibited preadipocyte proliferation. DHEAS did not significantly affect proliferation. *D*: the inhibitory effect of DHEA was not diminished when cells were coincubated with antagonists of the androgen receptor flutamide, the estrogen receptor faslodex, the glucocorticoid receptor RU-486, cortisol (F), and cortisone (E). Data represent means ± SE (*A*–*C*) or %control (*D*) obtained from at least 3 independent experiments. In *A*–*C*, *P < 0.05 and **P < 0.01. In *D*, *P < 0.05 vs. control and #*P* < 0.05 vs. DHEA.

**Fig. 4.**
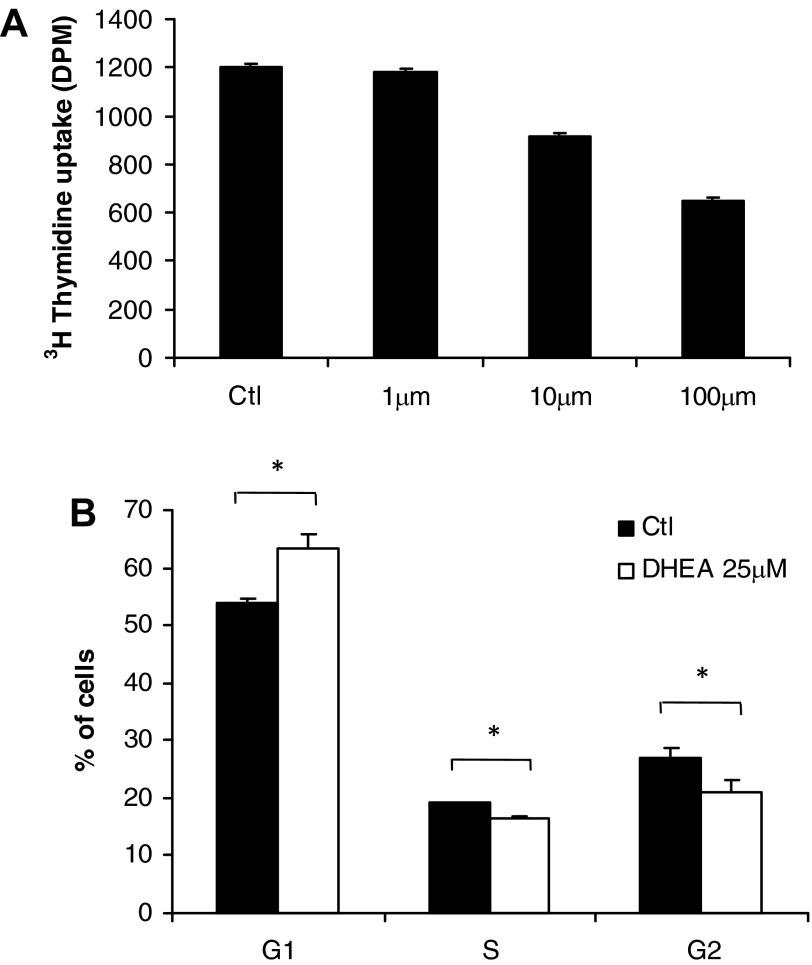
DHEA results in inhibition of preadipocyte proliferation via growth arrest in the G1 phase. *A*: incubation of human primary preadipocytes with DHEA (0–100 μM) inhibited proliferation, as shown by decreased [^3^H]thymidine uptake (DHEA; ≥10 μM). *B*: treatment of subconfluent proliferating Chub-S7 cells with 25 μM DHEA significantly increased the no. of cells in G1 phase [63 vs. 54% in untreated control (Ctl) cells] and decreased the no. of cells in S phase (16 vs. 27%) and G2 phase (19 vs 21%). Data represent means ± SE derived from 5 independent triplicate experiments. *P < 0.05. DPM, disintegrations per minute.

Because the acquisition of adipose mass in vivo requires both preadipocyte proliferation and differentiation, we examined the effects of DHEA on both of these processes. DHEA (≥1 μM) caused a dose-dependent inhibition of Chub-S7 proliferation, as assessed by thymidine incorporation assays (Fig. 3*A*). Androstenediol (≥10 μM) had less effect, and DHEAS had no effect at all on Chub-S7 proliferation (Fig. 3, *B* and *C*). Coincubation with the estrogen receptor antagonist faslodex and the androgen receptor antagonist flutamide failed to reverse the inhibitory effect of DHEA on Chub-S7 proliferation (Fig. 3*D*), indicating that DHEA does not act via these receptors. Furthermore, coincubation with either cortisol or cortisone did not reverse the DHEA effect (Fig. 3*D*). Interestingly, coincubation with the glucocorticoid receptor antagonist RU-486 resulted in an additive inhibitory effect on Chub-S7 proliferation (Fig. 3*D*). Experiments using human primary subcutaneous preadipocytes confirmed that DHEA (≥1 μM) inhibited preadipocyte proliferation (Fig. 4*A*).

We hypothesized that the inhibitory effect of DHEA was due to aberrant cell cycle progression and carried out FACS cell cycle analysis of Chub-S7 cells. Results revealed that DHEA caused a significant increase in the G1 phase while concurrently significantly reducing the proportion of cells in G2 and S phase (Fig. 4*B*).

### Effects of DHEA on Preadipocyte Differentiation

**Fig. 5.**
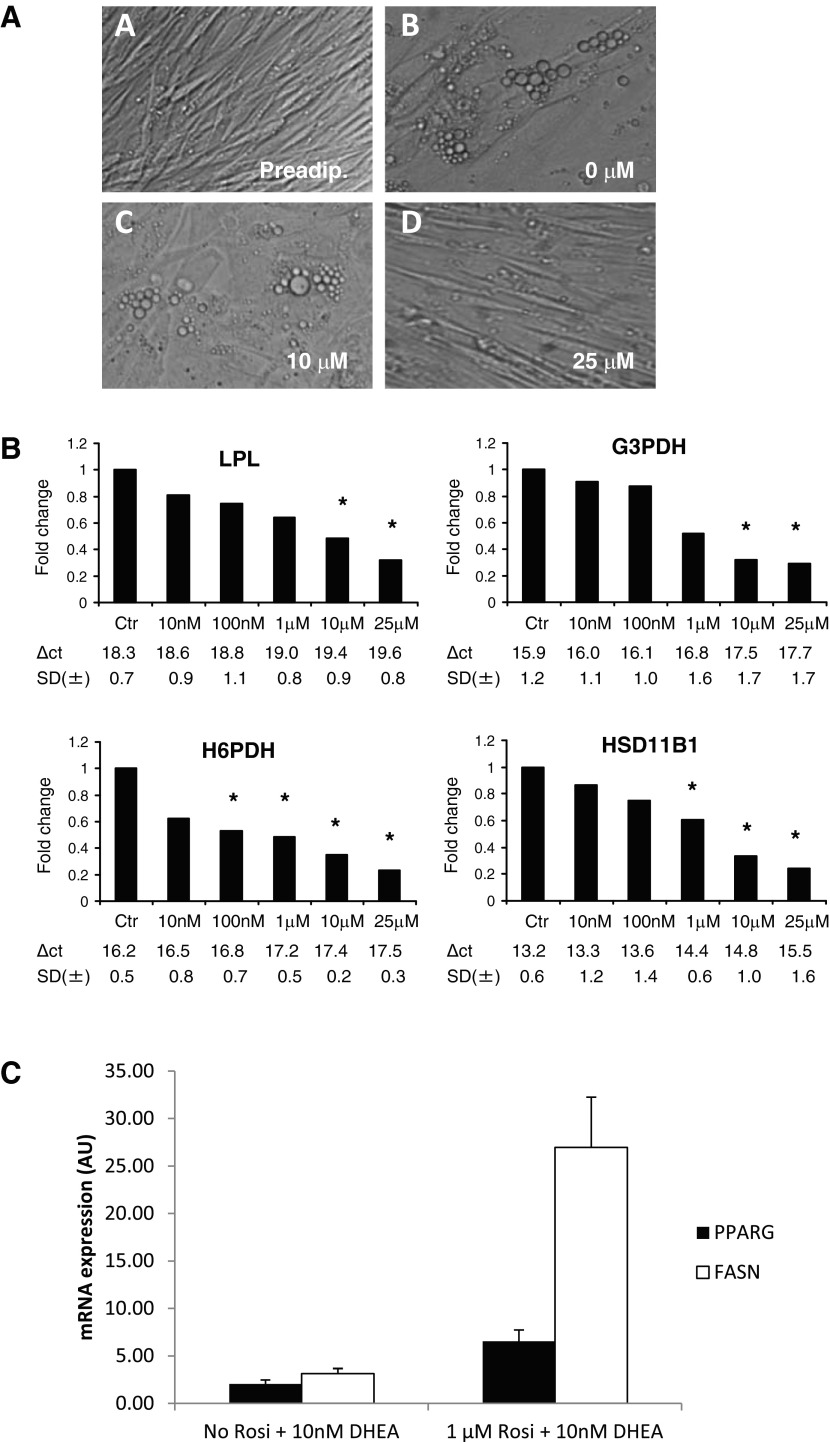
DHEA inhibits preadipocyte differentiation but not *PPARG* expression. *A*: morphological changes during differentiation of Chub-S7 cells (*image A*) are attenuated by DHEA treatment. Confluent Chub-S7 preadipocytes were differentiated in chemically defined media for 21 days in the presence of DHEA (0–50 μM). Control cells (*image B*) displayed a differentiated morphology with the acquisition of lipid droplets and the loss of a fibroblast-like appearance. Cells treated with 10 μM DHEA (*image C*) did not appear to differ morphologically from control cells. In contrast, cells treated with 25 μM DHEA (*image D*) appeared more fibroblast like than control cells, with an elongated shape, and contained much fewer lipid droplets. *B*: Chub-S7 cells were differentiated in the presence of DHEA (0–25 μM) and 500 nM cortisone for 21 days. mRNA expression analysis is presented at the time point where the most significant effect of DHEA was observed: *LPL* and *H6PDH*, *day 14*; *G3PDH* and *11β-HSD1*, *day 21*. mRNA expression was determined by quantitative PCR analysis and expressed as fold change of control. Data was obtained from at least 3 independent experiments *C*: Chub-S7 cells were differentiated for 14 days in the presence of DHEA (10 nM) and with or without the addition of 1 μM rosiglitazone. Expression of white adipose tissue differentiation marker *FASN* increased with the addition of rosiglitazone, but DHEA did not result in inhibition of *PPARG* expression. Data obtained from 3 independent experiments; differences in expression did not reach statistical significance. *P < 0.05.

The effect of DHEA on preadipocyte differentiation was assessed by observing cell morphology and mRNA analysis of the expression of differentiation markers. Treatment with 25 μM DHEA resulted in the cells appearing more fibroblast like, with less lipid accumulation, whereas no morphological differences were observed at lower concentrations (Fig. 5*A*). DHEA coincubated with cortisone caused a dose-dependent inhibition of the mRNA expression of early and terminal differentiation markers *LPL* (≥10 μM) and *G3PDH* (≥10 μM), respectively (Fig. 5*B*). Androstenediol treatment resulted in less pronounced retardation of gene expression (*LPL*, *G3PDH*, ≥25 μM; data not shown), and DHEAS did not demonstrate any effect (data not shown).

To test whether DHEA interferes with the PPARγ pathway, especially in view of the use of rosiglitazone in the Chub-S7 differentiation media, we differentiated Chub-S7 cells in the presence or absence of rosiglitazone in media containing 10 nM DHEA. Expectedly, addition of rosiglitazone resulted in an almost ninefold induction of fatty acid synthase (*FASN*), a marker of WAT differentiation (Fig. 5*C*). Furthermore, the presence of DHEA did not inhibit *PPARG* expression (Fig. 5*C*).

### Effects of DHEA on Adipocyte 11β-HSD1 Expression and Activity

**Fig. 6.**
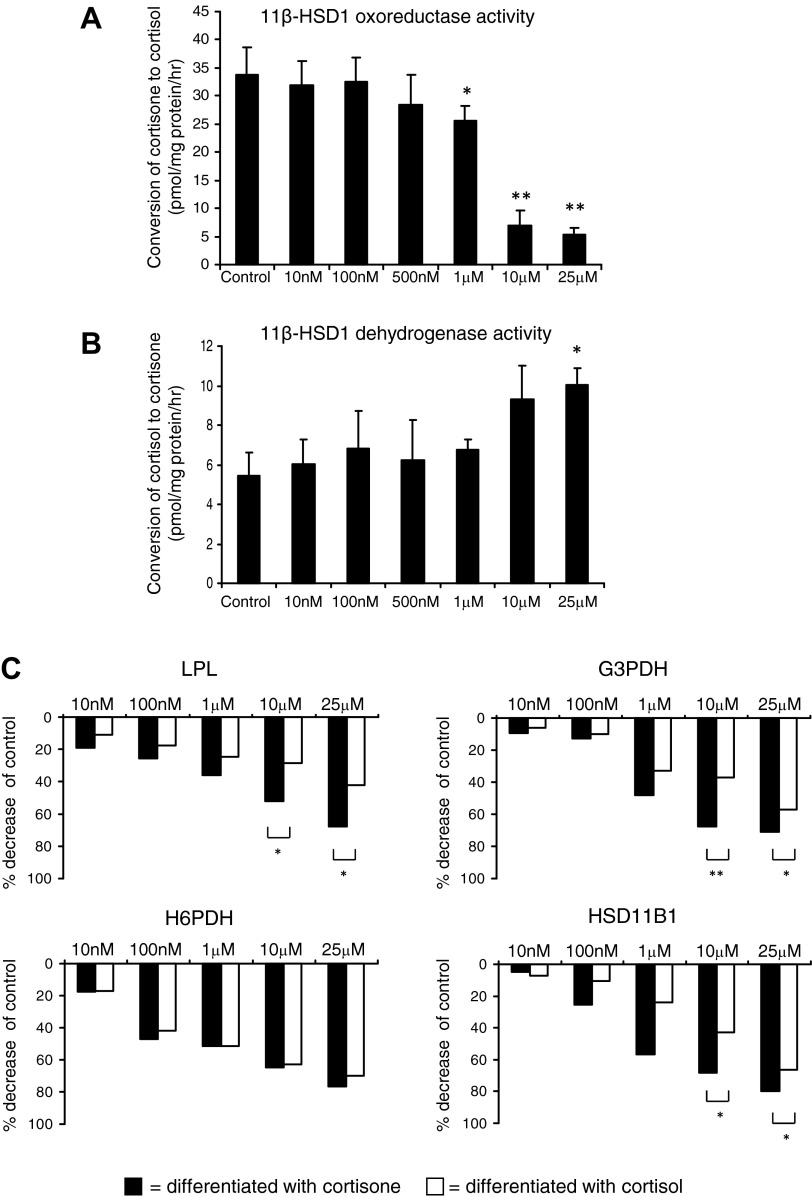
DHEA attenuates preadipocyte differentiation via inhibition of 11β-hydroxysteroid dehydrogenase type 1 (11β-HSD1) oxoreductase activity. *A* and *B*: cells differentiated for 21 days in the presence of DHEA (0–25 μM) were incubated with serum-free DMEM containing 100 nM cortisone and 50,000 cpm/ml [^3^H]cortisone for 3 h. Individual well protein concentrations were calculated and used as an internal control. DHEA (≥1 μM) significantly inhibited 11β-HSD1 oxoreductase activity (*A*) and increased dehydrogenase activity (*B*). Data are expressed as means ± SE of 3 independent triplicate experiments. *C*: Chub-S7 cells were differentiated in the presence of DHEA (0–25 μM) and 500 nM cortisone (black bars) or 500 nM cortisol (open bars). mRNA expression analysis is presented at the time point where the most significant effect of DHEA coincubated with cortisone was observed: *LPL* and *H6PDH*, *day 14*; *G3PDH* and *11β-HSD1*, *day 21*. mRNA expression was determined by quantitative PCR and expressed as %decrease in control. Data were obtained from 3 independent experiments. Statistical analysis was performed on ΔC_T_ values. DHEA (≥10 μM), when coincubated with inactive cortisone, significantly inhibited *LPL*, *G3PDH*, and *11β-HSD1* expression to a greater extent than when coincubated with active cortisol. In contrast, there was no significant difference in *H6PDH* expression between treatments. *P < 0.05 and **P < 0.01 vs. control.

DHEA treatment inhibited expression of the key glucocorticoid-regulating genes *H6PDH* (≥100 nM) and *HSD11B1* (≥1 μM) in differentiating preadipocytes in a dose-dependent manner (Fig. 5*B*). In keeping with this finding, DHEA treatment (≥1 μM) resulted in a marked reduction in 11β-HSD1 oxoreductase activity (≥1 μM; Fig. 6*A*) and a concurrent increase in dehydrogenase activity at the highest DHEA dose used (25 μM DHEA; Fig. 6*B*) in differentiated adipocytes. Again, DHEAS had no effect, and androstenediol had a lesser effect (data not shown).

### Functional Significance of DHEA Mediated Inhibition of 11β-HSD1

We postulated that the inhibition of 11β-HSD1 activity may account for the observed inhibitory effect of DHEA on preadipocyte differentiation. To test this hypothesis, we analyzed the effect of DHEA on adipocytes differentiated in the presence of the active glucocorticoid cortisol, negating the requirement for 11β-HSD1 activity for differentiation. In these cultures, the inhibitory effect of DHEA on mRNA expression of differentiation markers was significantly diminished (Fig. 6*C*). At 10 μM DHEA, *LPL* expression was reduced by 52% in differentiation assays with cortisone compared with 28% when coincubated with cortisol; similar effects were observed for *G3PDH* (DHEA + cortisone 68%, DHEA + cortisol 37%) and *HSD11B1* (DHEA + cortisone 68%, DHEA + cortisol 43%) (Fig. 6*C*). Interestingly, coincubation with cortisol did not significantly alter the level of DHEA-induced inhibition of H6PDH expression (Fig. 6*C*).

### Effects of DHEA on Adipocyte Glucose Uptake

**Fig. 7.**
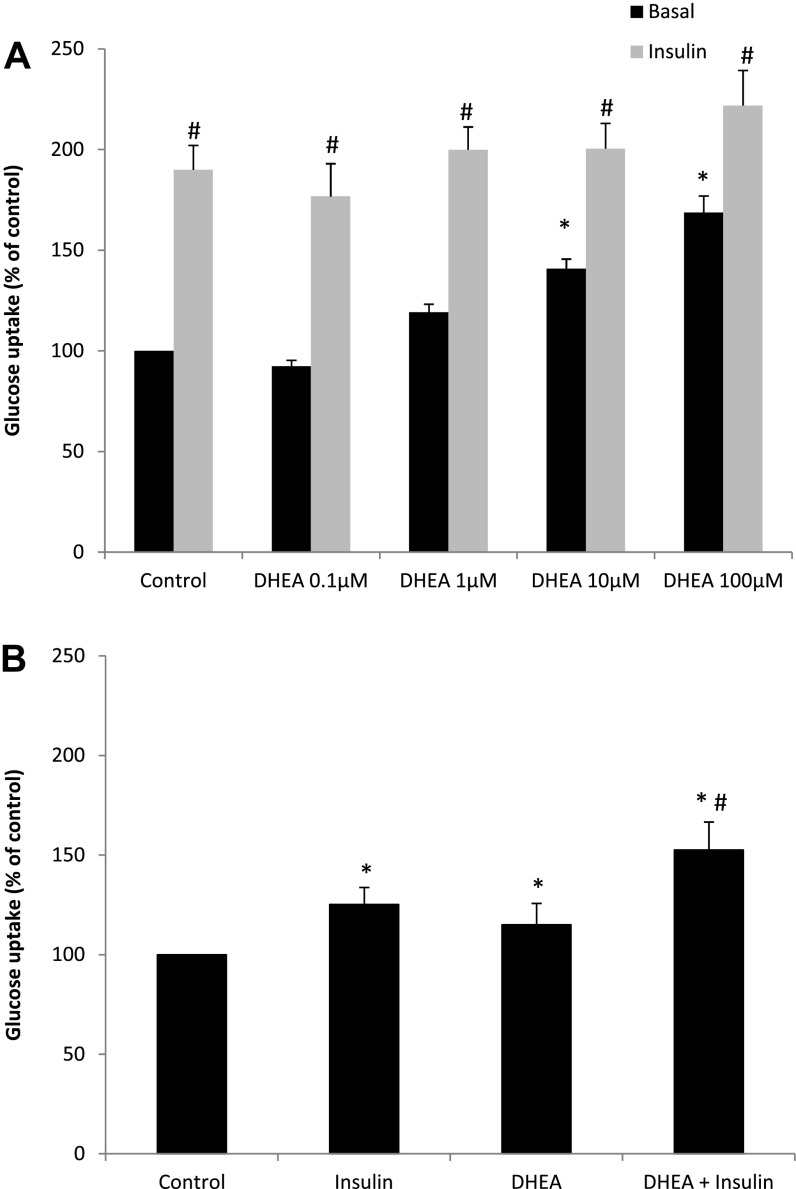
DHEA increases basal but not insulin-dependent glucose uptake in adipocytes. *A*: in mature Chub-S7 adipocytes, DHEA (≥1 μM) significantly increased basal glucose uptake (black bars) but not insulin-stimulated glucose uptake (20 nM; gray bars). *B*: in human primary adipocytes, insulin increased glucose uptake to 125 ± 8.6% (*P* < 0.05) and 25 μM DHEA to 115 ± 10.7% (*P* < 0.05) compared with basal. Addition of insulin to DHEA increased glucose uptake to 133 ± 14.0% compared with DHEA alone (*P* < 0.05). Data are presented as %control from 5 experiments performed in triplicate [Chub-S7 control DPM = 5,257 (*A*); human primary adipocytes control DPM = 5,212 (*B*)]. *P < 0.05 vs. control; #*P* < 0.05 vs. DHEA treatment.

DHEA significantly increased basal glucose uptake (*P* < 0.05 for 10 and 100 μM DHEA; Fig. 7*A*) in mature Chub-S7 adipocytes. Interestingly, insulin-stimulated glucose uptake was not affected by the addition of DHEA (Fig. 7*A*). We confirmed the validity of this result employing human primary subcutaneous preadipocytes, which demonstrated comparable results using a 25-μM DHEA concentration (Fig. 7*B*). Compared with basal, insulin increased glucose uptake to 125 ± 8.6% (*P* < 0.05) and DHEA to 115 ± 10.7% (*P* < 0.05). Addition of insulin to DHEA increased glucose uptake to 133 ± 14.0% compared with DHEA alone (*P* < 0.05).

## DISCUSSION

We have validated the Chub-S7 cell model and have confirmed that it is representative of human subcutaneous white adipose tissue, as described before (6). Thiozolidinediones (TZDs) are known to induce a BAT-like phenotype in human preadipocytes (15). We investigated whether the use of the TZD rosiglitazone during the Chub-S7 differentiation process could have altered the cellular phenotype. Our findings indicate that TZD treatment of Chub-S7 cells does not result in any relevant *UCP-1* expression, which was similar to the extremely low levels found in other non-BAT tissues.

We have found that DHEA exerts antiglucocorticoid action at several levels in adipose tissue, including adipogenesis and basal glucose uptake. Previous studies have shown that the expression and activity of 11β-HSD1 is inhibited by DHEA in murine adipocytes (2, 43) and rat liver (22), suggesting a mechanistic link between DHEA and glucocorticoids, but the functional significance of this finding was previously unknown. Here, we have shown that inhibition of 11β-HSD1 contributes significantly to attenuation of preadipocyte differentiation mediated by DHEA.

We have demonstrated a clear inhibitory effect of DHEA on preadipocyte differentiation, characterized by attenuation of the expression of differentiation markers, morphological changes, and lipid accumulation. This is in line with previous studies alluding to an inhibitory effect of DHEA on preadipocyte differentiation. Lea-Curie et al. (33) observed that DHEA attenuated triacylglycerol accumulation in differentiating 3T3-L1 cells. Kajita et al. (29) have reported that DHEA treatment of rodents decreased the expression of PPARγ, a regulator of adipocyte differentiation, in the adipose tissue of these animals. More recently, it was shown that DHEA reduces body weight and epididymal fat in a murine model of obese type 2 diabetes (18). Interestingly, it was shown that although DHEA does not affect differentiation of human primary subcutaneous preadipocytes, it has inhibitory effects on proliferation and differentiation of omental preadipocytes (39). However, all of these previous studies have failed to provide clear mechanistic insights into the effects of DHEA.

We have shown that the inhibitory effect of DHEA on preadipocyte differentiation is via an antiglucocorticoid mechanism. It is well characterized that glucocorticoids, reactivated locally by the oxoreductase activity of 11β-HSD1, play a vital permissive role in inducing preadipocyte differentiation (7, 21, 24, 48). In addition, the selective inhibition of 11β-HSD1 in human primary preadipocytes and Chub-S7 cells impairs the differentiation of these cells (6). We have demonstrated that DHEA attenuates the local regeneration of glucocorticoids in adipocytes and have shown this is via two concerted mechanisms: attenuation of the induction of 11β-HSD1 during differentiation and inhibition of expression of H6PDH, the enzyme that regenerates the cofactor NADPH that is required for 11β-HSD1 oxoreductase activity (25). We have demonstrated that inhibition of expression of these genes has physiological significance, inhibiting the local amplification of glucocorticoids resulting in the attenuation of the differentiation of preadipocytes. Furthermore, via coincubation assays with the active glucocorticoid cortisol, we have demonstrated conclusively that it is via 11β-HSD1, at least in part, that DHEA mediates the inhibition of adipocyte differentiation. However, it is clear that DHEA acts additionally via glucocorticoid-independent mechanisms since coincubation with cortisol still resulted in inhibition of preadipocyte differentiation, albeit significantly diminished.

A previous study employing a murine model has suggested that DHEA-dependent effects may be mediated via the androgen receptor (18). This is in contrast to our findings, as we could not demonstrate any androgen receptor-mediated DHEA effects in human preadipocytes with no evidence of the reversal of the inhibitory effects of DHEA when coincubating with the androgen receptor antagonist flutamide.

It is as yet unknown whether DHEA inhibits 11β-HSD1 gene expression directly or via a regulator of 11β-HSD1 expression such as the adipogenic gene C/EBPα (1), which may account for the remaining inhibitory effect of DHEA upon coincubation with cortisol. Others have shown that murine adipocyte DHEA acts as a noncompetitive inhibitor of 11β-HSD1 independently of transcriptional control (43).

One limitation of our study is the use of TZDs during the differentiation process of our model cell line. TZDs are known to promote adipogenesis via PPARγ-mediated pathways, whereas the PPARγ gene locus *PPARG* is regulated by C/EBPs and the glucocorticoid receptor (13). Although it is clear that TZDs are important differentiation determinants in our model cell line, we could show that DHEA does not inhibit *PPARG* expression in our model. Hence, it seems unlikely that the observed inhibitory effects of DHEA on differentiation are due to interference with the TZD/PPARγ pathway. Clearly, more studies exploring the exact mechanistic effects of DHEA on adipocyte differentiation are warranted.

The effect of DHEA is specific, as neither DHEAS nor the principal metabolite of DHEA in adipocytes, androstenediol (19), produced comparable inhibitory effects. Conversion of DHEA in preadipocytes is negligible, and in support of specificity, detection of OATP-D, an influx transporter of DHEAS (38), suggests that the lack of effect of DHEAS is not due to its inability to enter the cell.

In addition to the inhibitory effect of DHEA on preadipocyte differentiation, we have demonstrated that DHEA significantly attenuates preadipocyte proliferation and increases basal adipocyte glucose uptake. Some of these findings have been described previously, utilizing murine models (1, 18, 43). However, because mice do not produce DHEA physiologically, the validity of these results to humans was previously unclear. Our study demonstrates conclusively that DHEA has similar effects on adipocyte biology in murine and human cells, suggesting that DHEA may have beneficial effects in vivo. Further human in vivo studies are warranted, especially since DHEA effects on different metabolic tissues have been described.

Our observation that DHEA results in preadipocyte proliferation through arrest in the G1 phase of the cell cycle is consistent with earlier murine studies utilizing 3T3-L1 cells (20, 32, 34) and human preadipocytes (39). This effect is in contrast to glucocorticoids, which are known to stimulate subcutaneous preadipocyte proliferation in a depot-specific manner (3). From a mechanistic point of view, the DHEA-mediated inhibition of preadipocyte proliferation appears to be independent of the androgen and estrogen receptor-mediated action. Moreover, the finding that, in contrast to preadipocyte differentiation, replacement of inactive cortisone by cortisol does not result in an attenuation of the DHEA effect suggests an antiproliferative mechanism that is independent of 11β-HSD1. This is further supported by the observed additive effect after coincubation with the glucocorticoid receptor antagonist RU-486 on preadipocyte proliferation. However, it must be noted that RU-486 not only is a glucocorticoid receptor antagonist but has additional actions, e.g., at the progesterone receptor. Thus we cannot exclude other mechanisms resulting in the observed additive effects.

We have shown DHEA to stimulate basal glucose uptake, mimicking the action of insulin, consistent with the amelioration of hyperglycemia and insulin resistance observed upon DHEA treatment in vivo (47). Again, this effect of DHEA opposes that of glucocorticoids, which induce insulin resistance in preadipocytes (41, 46). The underlying mechanisms by which glucocorticoids modulate insulin signaling remain unclear but appear to involve the conventional protein kinase C (PKC) (27, 30). In direct contrast, it has been demonstrated utilizing rat adipocytes that DHEA may mimic insulin action via PI3K and atypical PKC activation (26) independent of the insulin receptor or Akt activation, resulting in increased translocation of the glucose transporters GLUT1 and GLUT4 to the plasma cell membrane (37). These findings are consistent with the rapid stimulatory effect of DHEA on basal glucose uptake observed in this study, which suggests that DHEA is acting via a nongenomic mechanism. However, further studies are required to elucidate the exact mechanism underlying the observed DHEA effects on glucose uptake.

Our findings indicate the likelihood of favorable metabolic effects of DHEA in vivo as it opposes the effects of glucocorticoids by distinct mechanisms. The adverse metabolic effects of glucocorticoids are well characterized and exemplified in patients with Cushing's syndrome (8). Transgenic mice overexpressing 11β-HSD1 under control of the *aP2* promoter display the full-blown metabolic syndrome, including increased adiposity (36). Conversely, knockdown of 11β-HSD1 in mice results in protection from high-fat diet-induced development of the metabolic syndrome, including reduced adiposity and improved insulin sensitivity (31). The inhibition of 11β-HSD1 activity or activation of opposing pathways could have beneficial effects in vivo. Indeed, selective inhibition of 11β-HSD1 activity is emerging as an exciting, novel therapeutic approach in type 2 diabetes and the metabolic syndrome (40).

Our findings establish that DHEA inhibits human subcutaneous adipogenesis and increases glucose uptake in vitro, actions that oppose those of glucocorticoids. Our findings are in keeping with the significant increase in glucose sensitivity and reduction in adiposity reported by Villareal and Holloszy (47) in elderly subjects treated with 50 mg/dl DHEA for 6 mo. However, antiglucocorticoid action of DHEA is metabolically counteracted by its androgenic action, which can also yield unfavorable metabolic consequences. Further studies to elucidate the effects of DHEA on human metabolism and adipose tissue function are warranted to unravel the regulatory mechanisms underlying the delicate balance between glucocorticoid and androgen action.

## GRANTS

This research was supported by the Medical Research Council UK (Ph.D studentship to J. C. McNelis, Senior Clinical Fellowship G0802765 to J. W. Tomlinson) and the Wellcome Trust (Project Grant No. 092283, to W. Arlt).

## DISCLOSURES

No conflicts of interest, financial or otherwise, are declared by the authors.

## AUTHOR CONTRIBUTIONS

J.C.M., K.N.M., L.L.G., P.M.S., J.W.T., and W.A. contributed to the conception and design of the research; J.C.M., K.N.M., L.L.G., and I.J.B. performed the experiments; J.C.M., K.N.M., L.L.G., and W.A. analyzed the data; J.C.M., K.N.M., L.L.G., I.J.B., P.M.S., J.W.T., and W.A. interpreted the results of the experiments; J.C.M., K.N.M., and L.L.G. prepared the figures; J.C.M. drafted the manuscript; J.C.M., K.N.M., L.L.G., P.M.S., J.W.T., and W.A. approved the final version of the manuscript; K.N.M., P.M.S., J.W.T., and W.A. edited and revised the manuscript.
